# Association of Pre-Operative Albuminuria with Post-Operative Outcomes after Coronary Artery Bypass Grafting

**DOI:** 10.1038/srep16458

**Published:** 2015-11-09

**Authors:** Lekha K. George, Miklos Z. Molnar, Jun L. Lu, Kamyar Kalantar-Zadeh, Santhosh K. G. Koshy, Csaba P. Kovesdy

**Affiliations:** 1 Division of Nephrology, Department of Medicine, University of Tennessee Health Science Center, Memphis, 956 Court Ave, Memphis, TN, 38163, USA; 2 Regional One Health, Memphis, 877 Jefferson Ave, Memphis, TN, 38103, USA; 3 Division of Nephrology, University of California, Irvine, 101 The City Drive, Orange, CA 92868, USA; 4 Division of Cardiology, Department of Medicine, University of Tennessee Health Sciences Center, Memphis, 956 Court Ave, Memphis, TN, 38163, USA; 5 Nephrology Section, Memphis Veterans Affairs Medical Center, Memphis, 1030 Jefferson Ave., Memphis TN 38104, USA

## Abstract

The effect on post-operative outcomes after coronary artery bypass graft(CABG) surgery is not clear. Among 17,812 patients who underwent CABG during October 1,2006-September 28,2012 in any Department of US Veterans Affairs(VA) hospital, we identified 5,968 with available preoperative urine albumin-creatinine ratio(UACR) measurements. We examined the association of UACR<30, 30–299 and >=300 mg/g with 30/90/180/365-day and overall all-cause mortality, and hospitalization length >10 days, and with acute kidney injury(AKI). Mean ± SD baseline age and eGFR were 66 ± 8 years and 77 ± 19 ml/min/1.73 m^2^, respectively. 788 patients(13.2%) died during a median follow-up of 3.2 years, and 26.8% patients developed AKI(23.1%-Stage 1; 2.9%-Stage 2; 0.8%-Stage 3) within 30 days of CABG. The median lengths of stay were 8 days(IQR: 6–13 days), 10 days(IQR: 7–14 days) and 12 days(IQR: 8–19 days) for groups with UACR < 30 mg/g, 30–299 mg/g and ≥300 mg/g, respectively. Higher UACR conferred 72 to 85% higher 90-, 180-, and 365-day mortality compared to UACR<30 mg/g (odds ratio and 95% confidence interval for UACR≥300 vs. <30 mg/g: 1.72(1.01–2.95); 1.85(1.14–3.01); 1.74(1.15–2.61), respectively). Higher UACR was also associated with significantly longer hospitalizations and higher incidence of all stages of AKI. Higher UACR is associated with significantly higher odds of mortality, longer post-CABG hospitalization, and higher AKI incidence.

Chronic kidney disease (CKD) is a risk factor for long-term adverse outcomes after coronary artery bypass graft (CABG) surgery. In recent years, albuminuria^1^ and the combination of low estimated glomerular filtration rate (eGFR) and albuminuria^2^ have been found to be significant risk factors in general population cohorts^3^ and in studies measuring short-term sequelae and long-term outcomes related to CKD and cardiac surgery^4^,^5^,^6^.

The effects of proteinuria could be particularly important in the post-operative period, due to its association with reduced coronary flow reserve and increased microvascular resistance^7^. Proteinuria was associated with higher mortality and end stage renal disease (ESRD) in a retrospective analysis examining 925 Taiwanese patients with all levels of renal function undergoing CABG, independent of pre-operative eGFR and post-operative acute kidney injury (AKI)^8^. Risk scores for post-CABG outcomes have emphasized the importance of pre-operative eGFR^9^,^10^,^11^ and CKD with dipstick proteinuria^12^ in predicting the risk of post-CABG AKI. However, these studies are limited by relative short-term follow up. It is also unknown if the same relationship exists in patients with eGFR ≥60 ml/min/1.73 m^2^ and if it would be applicable to a larger population in a different geographic area.

We hence examined the association of pre-operative albuminuria with short- and long-term mortality and length of hospital stay, and also with post-operative AKI in a large cohort of US veterans undergoing CABG. We hypothesized that the degree of albuminuria is associated with proportionally higher risk of the studied outcomes.

## Results

### Baseline characteristics

The mean ± SD age of the cohort at baseline was 66 ± 8 years, 85% and 10% of patients were white and black, respectively, 88% of the patients were diabetic and the mean baseline eGFR was 77 ± 19 ml/min/1.73 m^2^. Baseline characteristics of patients categorized by UACR status are shown in Table 1. The level of eGFR, and the prevalence of diabetes, CHF, stroke, peripheral arterial disease were progressively higher in patients with higher UACR. Use of ACEI or ARB pre-operatively or during in-hospital stay was higher in patients with higher UACR. Compared to the analytic cohort (n = 5,968), patients excluded because of missing UACR (n = 11,844) had lower BMI, and a lower proportion of diabetes and hypertension at the time of study entry (results not shown).

### Mortality

**Figure 1:**
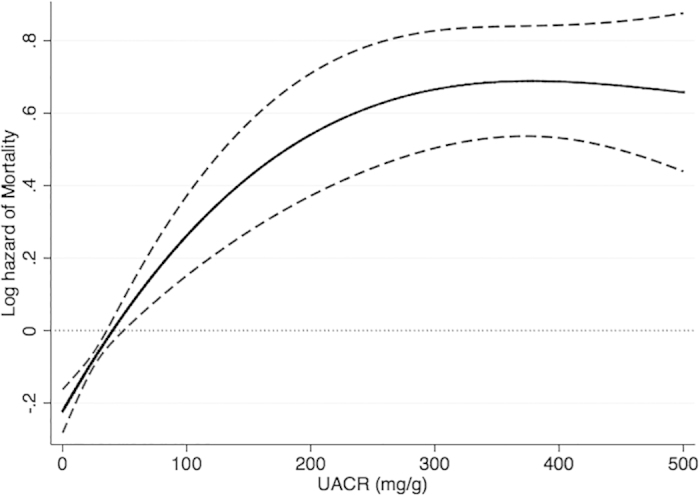
Association of UACR level with all-cause mortality in the Cox proportional hazard model.

Out of 5,968 patients 13.2% (n = 788) died during a median 3.2 years of follow-up. There were 417 deaths (10.8%, mortality rate 32 [29–35]/1000 patient-years) in the UACR<30 mg/g group; 266 deaths (15.9%, 50 [44–57]/1000 patient-years) in the UACR 30–299 mg/g group and 105 deaths (23.6%, 79 [66–96]/1000 patient-years) in the UACR ≥300 mg/g group. There was 38% higher (HR: 1.38, 95% CI: 1.18–1.62) risk of mortality in patients with UACR 30–299 mg/g and more than double (HR: 2.08, 95% CI: 1.64–2.62) risk of mortality in patients with UACR ≥300 mg/g compared to patients with UACR<30 mg/g. The association between UACR as continuous variable and overall mortality is shown in Fig. 1.

**Figure 2:**
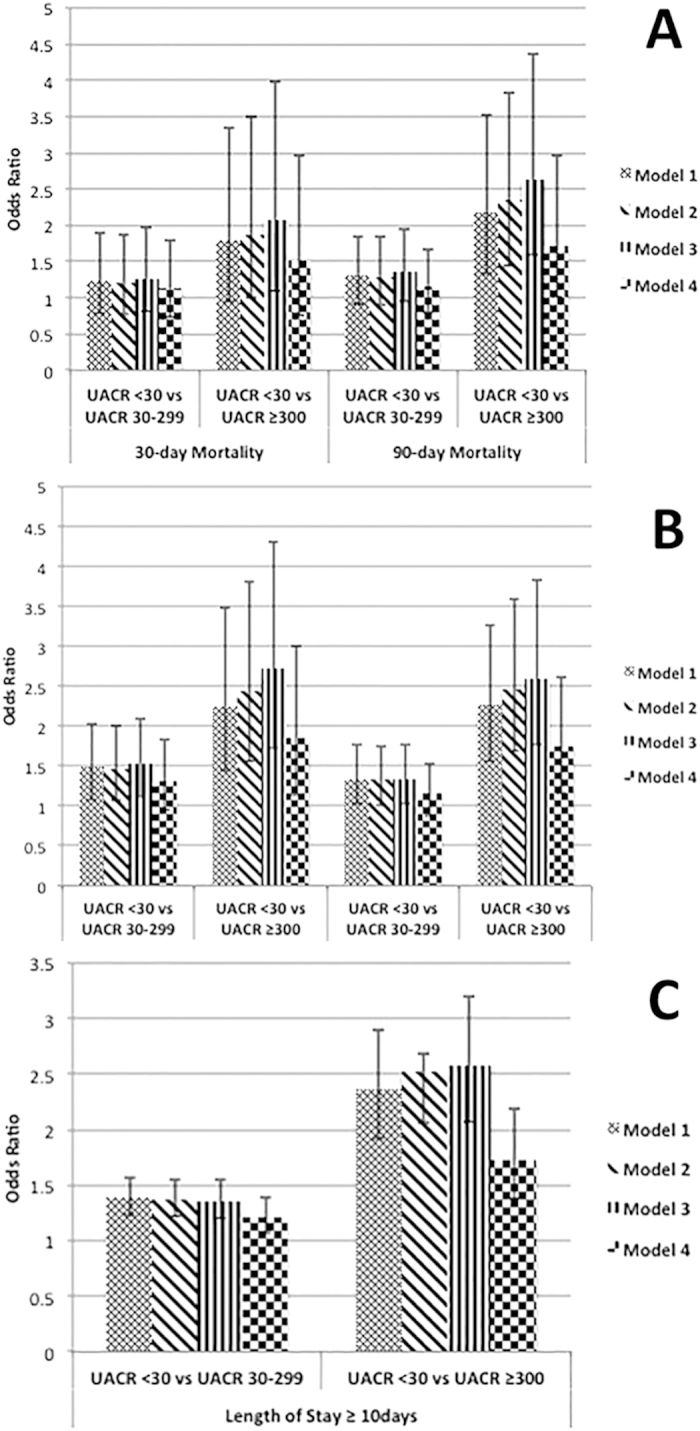
Association of UACR level with outcomes in different levels of adjustment. (Panel **A**) for 30- and 90-day mortality, (Panel **B**) for 180- and 365-day mortality and (Panel **C**) Length of hospital stay. Model 1 – unadjusted; Model 2 – model 1 adjusted for age, gender, race/ethnicity; Model 3- model 2 adjusted for SBP, DBP, Preoperative ACE inhibitor and statin use, In-hospital ACE inhibitor and statin use; Model 4 – model 3 adjusted for eGFR, cholesterol, DM, CHF, CCI. All displayed as odds ratio and 95% confidence interval.

The number of patients and the risk of 30/90/180/365-day mortality are shown in Table 2. Figure 2A,B display the effect of sequential adjustment on the association of albuminuria with post-operative mortality. Compared to patients with UACR <30 mg/g, patients with UACR 30–299 mg/g had similar 30/90/180/365-day mortality risk. Patients with UACR ≥300 mg/g had significantly higher risk for 90/180/365-day mortality compared to patients with UACR<30 mg/g (Table 2), but similar 30-day mortality risk (HR: 1.50; 95% CI: 0.75–2.97). Similar trends were found in propensity-matched cohort analyses (Supplementary Table S1).

### Length of hospital stay

The median length of stay for the overall cohort was 9 days (IQR: 6–14 days). The median lengths of stay were 8 days (IQR: 6–13 days), 10 days (IQR: 7–14 days) and 12 days (IQR: 8–19 days) for groups with UACR < 30 mg/g, 30–299 mg/g and ≥300 mg/g, respectively. After adjustment for all confounders, the predicted mean lengths of stay were 9.1 ± 2.2 days, 10.6 ± 2.6 days and 12.3 ± 3.0 days in patients with UACR < 30 mg/g, 30–299 mg/g and ≥300 mg/g, respectively.

Out of a total of 5,686 patients, 3,372 (59.3%) patients were hospitalized for <10 days after CABG, whereas 2,314 patients had a prolonged hospitalization of ≥10 days. 36.9% (1,353/3,667), 44.8% (713/1,591) and 57.9% (248/428) of patients with UACR <30 mg/g, 30–299 mg/g and ≥300 mg/g, respectively, were hospitalized for ≥10 days. Figure 2C displays the association of proteinuria with post-operative length of hospital stay after sequential adjustments. In fully adjusted models, compared to patients with UACR<30 mg/g, patients with UACR 30–299 mg/g had 22% higher (OR: 1.22, 95% CI: 1.07–1.39), while patients with UACR ≥300 mg/g had 73% (OR: 1.73, 95% CI: 1.38–2.18) higher risk of being hospitalized for ≥10 days after CABG (Table 3). Similar results were found in propensity-matched cohort analyses (Supplementary Table S1).

### Acute kidney injury

5,359 patients had pre-operative and post-operative inpatient serum creatinine levels measured, of which 73.2% (3,923) patients did not develop AKI, whereas 23.1% (1,239) developed stage 1 AKI, 2.9% (155) developed stage 2 AKI and 0.8% (42) developed stage 3 AKI. The proportion of patients who did not develop AKI was 76.1% (2,634/3,463), 70.1% (1,048/1,495) and 60.1% (241/401) in patients with UACR < 30 mg/g, 30–299 mg/g and ≥300 mg/g, respectively.

Compared to patients with UACR<30 mg/g, patients with UACR 30–299 mg/g and patients with UACR ≥300 mg/g had significantly higher multivariable adjusted risk of each AKI stage (Table 3). Similar results were found in propensity-matched cohort analyses (Supplementary Table S1).

## Discussion

We describe an independent association between preoperative albuminuria and 90/180/365 and overall mortality, length of hospitalization and AKI following CABG in this cohort with baseline eGFR ≥60 ml/min/1.73 m^2^. These associations were independent of eGFR level and comorbid conditions. The various outcomes showed a graded association with increasing severity of albuminuria.

Post-operative AKI after CABG is common and it is more frequently developing if the patient is operated more than 24 hours after cardiac catheterization^13^. Similar to our findings, in cohorts of CKD patients preoperative increased levels of proteinuria were associated with higher risk of mortality in patients who developed AKI after cardiac surgery^8^,^14^. In our study, the 90-, 180- and 365-day mortality was higher in patients with severe preoperative albuminuria. Similar to the study by Huang *et al.*^12^, we did not find an association between albuminuria and 30-day mortality. This could be explained by the closer attention devoted to patients in the immediate post-operative period, or by the longer time needed for pathophysiologic effects linked to higher albuminuria to manifest clinically following CABG. Alternatively, the lower number of events during such a short time period may result in decreased power to detect significant differences, in spite of a nominally higher risk of 30-day mortality. A lower power may also explain why we did not detect statistically significant associations between moderately higher albuminuria (UACR 30-299 mg/g) and mortality risk up to 365-days after CABG, even though the associations were nominally elevated, and overall mortality was significantly higher in this subgroup when considering the higher number of total deaths occurring throughout the entire follow-up period of our cohort.

Prior studies of patients with advanced CKD reported an association between proteinuria and the risk of postoperative AKI^5^,^8^,^12^,^15^,^16^. Our study shows that even in patients with eGFR ≥60 ml/min/1.73 m^2^ there is a higher risk of postoperative AKI, and a higher risk for more severe AKI associated with albuminuria after CABG surgery. Moreover, the magnitude of this risk increases with increasing degree of albuminuria and the presence of severe albuminuria (UACR>=300 mg/g) confers the highest risk of AKI in the post-operative period. Hence preoperative albuminuria should be an important variable in risk prediction models for postoperative AKI after CABG and it should be included to the future prediction score for AKI. A score post-operative atrial fibrillation was recently validated^17^, however no score with albuminuria was developed for AKI. It is important to identify such inexpensive preoperative markers of AKI so the utility of preventive measures aimed at correcting such risk factors during the perioperative period could be tested in randomized controlled clinical trials. The practical relevance of post-operative AKI is underscored by several large multicenter studies^14^,^18^,^19^ indicating that post-operative AKI is associated with increased risk of dialysis-requiring kidney failure, mortality, and increased hospital costs^20^,^21^.

A previous study indicated that the length of hospital and intensive care unit stay after cardiac surgery is increased in patients with preoperative proteinuria^4^. However it was not clear if including patients with advanced CKD influenced this association. Our study, which included patients with eGFR ≥60 ml/min/1.73 m^2^, showed that the length of hospital stay after CABG in patients with albuminuria was significantly higher. It is unknown what proportion of this increased length of stay can be attributed to the development of AKI, which was also increasingly seen in patients with higher preoperative proteinuria. Irrespective of the exact mechanisms involved, the increased duration of hospitalization could directly contribute to higher costs, and could expose patients to more hospitalization-associated adverse events such as nosocomial infections.

### Limitations of the study

This being an observational study, we can only report associations, and we cannot claim that UACR was indeed the cause of the worse clinical outcomes. Additionally, models could only be adjusted for confounders for which we had available data. Therefore, we cannot rule out residual confounding. The study population consisted of mostly male patients; hence, the results may not be generalizable to females. Because we did not have information about causes of death, we could not analyze associations with cause-specific mortality.

## Conclusion

Presence of preoperative albuminuria is associated with poorer post-operative outcomes in patients undergoing CABG. Albuminuria should be included as an important prognostic factor in risk prediction models for measuring clinical outcomes after CABG. The strength of association and degree of external validity of this risk associated with preoperative proteinuria on postoperative outcomes need to be further evaluated, and the effects of proteinuria-lowering strategies on post-CABG outcomes will need to be assessed in large prospective randomized controlled clinical trials.

## Methods

### Cohort Definition

**Figure 3:**
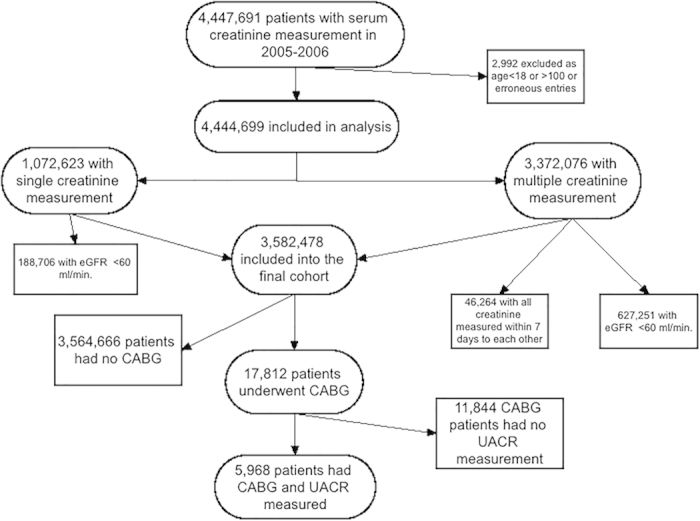
Flow chart of patient selection for the study.

Our study utilized data from a cohort study examining risk factors in patients with incident CKD (Racial and Cardiovascular Risk Anomalies in CKD (RCAV) study), as previously described^22^,^23^. Briefly, we used national Veterans Affairs (VA) Corporate Data Warehouse LabChem data files to identify veterans with eGFR of ≥60 ml/min/1.73 m^2^ during October 1, 2004-September 30, 2006^24^, calculated according to the Chronic Kidney Disease Epidemiology Collaboration (CKD-EPI) Equation^25^. The flowchart for patient selection for the study is displayed in Fig. 3. From among 3,582,478 patients with baseline eGFR ≥60 ml/min/1.73 m^2^ we identified 17,812 patients who underwent CABG between October 1, 2006 and September 28, 2012, identified from VA Inpatient and Outpatient Medical SAS Datasets using *International Classification of Diseases, Ninth Revision (ICD-9)* diagnostic and procedure codes and C*urrent Procedural Terminology (CPT)* codes. From these 17,812 patients, 5,968 patients, our final cohort, had urine albumin/creatinine ratio (UACR) measured during the pre-operative period. The UACR value nearest to the date of CABG was used for these analyses (median time between CABG and UACR measurement: 302 days (IQR: 115-628 days)). Socio-demographic characteristics, comorbid conditions and laboratory characteristics were obtained as previously described^26^,^27^,^28^,^29^. Information about age, gender and race were obtained through the VA Corporate Data Warehouse (CDW) and from Medicare through the VA-Medicare data merge project. Baseline characteristics were defined on or immediately preceding the date of CABG. The final cohort was divided into 3 groups according to UACR level: <30 mg/g, 30–299 mg/g and ≥300 mg/g.

### Outcomes

We defined three different outcomes: 1) 30-, 90-, 180-, and 365- day and overall mortality, 2) length of hospitalization, and 3) incidence and severity of AKI. Data on mortality was obtained from the VA Vital Status Files (VSF), which contain dates of death or last medical/administrative encounter from all sources in the VA system with sensitivity and specificity of 98.3% and 99.8%, respectively, as compared to the National Death Index as gold standard^30^. The length of hospitalization was analyzed as a continuous variable, and also by categorizing it as ≤10 and >10 days. AKI was sub-classified according to the Acute Kidney Injury Network creatinine-based criteria (no AKI, grade 1, grade 2, and grade 3)^31^,^32^, without considering urine output.

### Statistical Analysis

Descriptive analyses were performed and skewed variables were log-transformed. Data were summarized using proportions, means ( ± standard deviation, SD) or medians (interquartile range, IQR) as appropriate. Categorical variables were analyzed with chi-square test and continuous variables were compared using Student’s t-test or the Mann-Whitney U test, Kruskal-Wallis H test or ANOVA as appropriate. In all statistics, two-sided tests were used and the results were considered statistically significant if p was <0.05.

The association of UACR with 30/90/180/365-day mortality, with AKI and with hospitalization length >10 days was assessed using unadjusted and multivariable adjusted logistic regression models. To assess the association between UACR and overall mortality, we performed time-to-event analyses using Cox proportional regression models. In these analyses, patients were followed until death or were censored at the date of last healthcare or administrative visit, or on July 26, 2013. We also performed unadjusted and multivariable adjusted linear regression analyses to examine the association of UACR with length of hospital stay. Adjusted length of stay in each UACR category was predicted from multivariable regression models using Stata post-estimation commands. Non-linear associations were assessed using fractional polynomials and restricted cubic splines.

Models were adjusted sequentially for the following confounders based on a priori considerations: model 1: age, gender, race/ethnicity; model 2: model 1 variables and baseline systolic and diastolic blood pressure, administration of angiotensin converting enzyme inhibitors (ACEI) or angiotensin receptor blockers (ARB) pre-operatively and during the hospitalization, and administration of statins pre-operatively and during the hospitalization; model 3: model 2 variables and baseline eGFR, serum cholesterol, presence of diabetes mellitus, presence of congestive heart failure and the Charlson Comorbidity Index.

The propensity score method was used in a sensitivity analysis to account for baseline differences arising from dissimilarities in clinical and demographic characteristics of patients with and without albuminuria^33^. Variables associated with UACR were identified using logistic regression and were used to calculate propensity scores. STATA’s “psmatch2” command suite was used to generate propensity score-matched cohorts by a 1-to-1 nearest neighbor matching without replacement. Statistical analyses were performed using STATA MP Version 12 (STATA Corporation, College Station, TX). The study protocol was approved by the institutional review boards at the Memphis and Long Beach Veterans Affairs Medical Centers and at the University of Tennessee Health Sciences Center.

## Additional Information

**How to cite this article**: George, L. K. *et al.* Association of Pre-Operative Albuminuria with Post-Operative Outcomes after Coronary Artery Bypass Grafting. *Sci. Rep.* **5**, 16458; doi: 10.1038/srep16458 (2015).

## Supplementary Material

Supplemental table

## Figures and Tables

**Table 1:** Baseline characteristics of patients.

	Overall (N = 5968)	UACR <30 mg/gm (N = 3852)*	UACR 30-299 mg/gm (N = 1671)*	UACR ≥ 300 mg/gm (N = 445)*
Age-years (Mean ± SD)	65.6 ± 7.6	65.6 ± 7.6	66.0 ± 7.6	64.1 ± 7.4
eGFR-ml/min/1.73 m^2^ (Mean ± SD)	77.2 ± 18.8	79.3 ± 17.7	75.3 ± 19.2	66.2 ± 22.1
Male n (%)	5911 (99)	3815 (99)	1657 (99)	439 (99)
Race Caucasian n (%)	4977 (85)	3211 (85)	1404 (85)	362 (83)
Race African Americans n (%)	586 (10)	378 (10)	159 (10)	49 (11)
Race Hispanics n (%)	166 (3)	109 (3)	47 (3)	10 (2)
Race Other n (%)	155 (2)	97 (2)	41 (2)	17 (4)
Per capita Income (USD) (Median (25;75 percentile))	22,876 (13,159;32,154)	22,907 (13,211;32,228)	22,912 (13,241;32,050)	22,186 (12,035;31,733)
BMI-kg/m^2^ (Mean ± SD)	31.26 ± 5.97	31.07 ± 5.83	31.65 ± 6.11	31.44 ± 6.49
Hypertension n (%)	5813 (97)	3731 (97)	1643 (98)	439 (99)
SBP-mmHg (Mean ± SD)	133.9 ± 14.2	132.5 ± 13.5	135.5 ± 14.3	140.2 ± 17.5
DBP-mmHg (Mean ± SD)	73.7 ± 8.5	73.7 ± 8.4	73.4 ± 8.3	75.0 ± 9.2
Cholesterol-mg/dl (Mean ± SD)	163 ± 45	163 ± 43	162 ± 46	169 ± 56
DM n (%)	5252 (88)	3279 (85)	1550 (93)	423 (99)
CHF n (%)	1715 (29)	937 (24)	560 (34)	218 (49)
Stroke n (%)	1763 (30)	1087 (28)	517 (31)	159 (36)
Hemiplegia n (%)	23 (0.4)	12 (0.3)	9 (0.5)	2 (0.4)
Chronic Lung Disease n (%)	2588 (43)	1613 (42)	762 (46)	213 (48)
Dementia n (%)	33 (0.6)	20 (0.5)	12 (0.7)	1 (0.2)
PAD n (%)	893 (15)	497 (13)	289 (17)	107 (24)
Malignancy n (%)	928 (16)	581 (15)	274 (16)	73 (16)
Rheumatological Disease n (%)	120 (2)	77 (2)	34 (2)	9 (2)
Liver Disease n (%)	120 (2)	70 (2)	35 (2)	15 (3)
HIV infection n (%)	19 (0.3)	11 (0.3)	8 (0.5)	0 (0)
Charlson Index (Mean ± SD)	3.99 ± 1.83	3.75 ± 1.75	4.28 ± 1.83	4.96 ± 1.95
ACEI/ARB Pre Operative n (%)	4430 (74)	2769 (72)	1301 (78)	360 (81)
ACEI/ARB In-hospital n (%)	3814 (64)	2376 (62)	1120 (67)	318 (71)
Statin Pre-Operative n (%)	3821 (64)	2463 (64)	1301 (78)	305 (69)
Statin In-hospital n (%)	4574 (77)	2953 (77)	1281 (77)	318 (72)
Total Death (during the overall follow-up period) n (%)	788 (13)	417 (11)	266 (16)	105 (24)

Abbreviations: UACR-urine albumin creatinine ratio; SD-standard deviation; BMI- body mass index; SBP- systolic blood pressure; DBP- diastolic blood pressure; DM- diabetes mellitus; CHF- congestive heart failure; PAD- peripheral arterial disease; HIV- human immunodeficiency virus; ACEI- ACE inhibitor; ARB- angiotensin receptor antagonist.

**Table 2:** Association of UACR level with mortality.

	UACR <30 mg/g	UACR 30–299 mg/g	UACR ≥300 mg/g
Overall Mortality (HR, 95% CI) (N = 788)	1.0	1.38 (1.18–1.62)*	2.08 (1.64–2.62)*
30-Day Mortality (OR, 95% CI) (N = 102)	1.0	1.14 (0.73–1.79)	1.50 (0.75–2.97)
90-Day Mortality (OR, 95% CI) (N = 155)	1.0	1.15 (0.79–1.66)	1.72 (1.01–2.95)*
180-Day Mortality (OR, 95% CI) (N = 196)	1.0	1.32 (0.95–1.83)	1.85 (1.14–3.01)*
365-Day Mortality (OR, 95% CI) (N = 279)	1.0	1.16 (0.87–1.53)	1.74 (1.15–2.61)†

^*^p < 0.05.

^†^p < 0.01, compared to UACR <30 mg/g group. Abbreviations: UACR-urine albumin creatinine ratio; HR- hazard ratio; OR-odds ratio; CI-confidence interval; N- number of deaths during the respective time frames.

**Table 3:** Multivariable adjusted association of UACR level with length of hospital stay and AKI.

	UACR <30 mg/g (N = 3,852)	UACR 30-299 mg/g (N = 1,671)	UACR ≥300 mg/g (N = 445)
Length of Hospitalization ≥ 10 days (OR, 95% CI)	1.0	1.22 (1.07–1.39)*	1.73 (1.38–2.18)*
AKI (estimated incidence, % ± SD)			
Stage 1	21.1 ± 6.2	26.0 ± 7.0‡	33.0 ± 8.0†
Stage 2	2.5 ± 1.1	3.3 ± 1.4‡	5.0 ± 2.3†
Stage 3	0.7 ± 0.3	0.9 ± 0.4‡	1.4 ± 0.7†

^*^p < 0.001.

^†^p < 0.01.

^‡^p < 0.05, compared to UACR <30 mg/g group. Abbreviations: UACR-urine albumin creatinine ratio; AKI-acute kidney injury; OR-odds ratio; CI-confidence interval; SD-standard deviation.
