# Supplementary material for: Association of Pre-Operative Albuminuria with Post-Operative Outcomes after Coronary Artery Bypass Grafting
Source: Sci Rep. 2015 Nov 9;5:16458. doi: 10.1038/srep16458 (PMC4637927; doi:10.1038/srep16458)
Supplement: Supplemental table [file srep16458-s1.pdf]

SREP-15-21556.R1

Association of Pre-Operative Albuminuria with Post-Operative Outcomes after Coronary Artery Bypass Grafting

Lekha K. George, MD[a,b]; Miklos Z. Molnar, MD, PhD[a]; Jun L. Lu, MD[a]; Kamyar Kalantar-Zadeh, MD, MPH, PhD[c]; Santhosh K. G. Koshy, MD, MBA[b,d] and Csaba P. Kovesdy MD[a,e]

[a]Division of Nephrology, Department of Medicine, University of Tennessee Health Science Center, Memphis, TN (956 Court Ave, Memphis, TN, 38163, USA); [b]Regional One Health, Memphis, TN (877 Jefferson Ave, Memphis, TN, 38103, USA); [c]Division of Nephrology, University of California, Irvine, CA (101 The City Drive, Orange, CA 92868, USA); [d]Division of Cardiology, Department of Medicine, University of Tennessee Health Sciences Center, Memphis, TN (956 Court Ave, Memphis, TN, 38163, USA); [e]Nephrology Section, Memphis Veterans Affairs Medical Center, Memphis, TN (1030 Jefferson Ave., Memphis TN 38104, USA)

SUPPLEMENT TABLE

Supplementary Table S1: Association between severity of proteinuria and post-CABG outcomes in the propensity-matched cohort

	UACR <30 mg/g (N=1,984)	UACR ≥30 mg/g (N=1,984)
30-Day Mortality (OR, 95%CI)	1.0	1.23 (0.76-1.99)
90-Day Mortality (OR, 95%CI)	1.0	1.27 (0.87-1.86)
180-Day Mortality (OR, 95%CI)	1.0	1.47 (1.04-2.06) ‡
365-Day Mortality (OR, 95%CI)	1.0	1.28 (0.96-1.69)
Length of Hospitalization >10 days (OR, 95%CI)	1.0	1.28 (1.12-1.45)*
AKI (estimated incidence, %±SD)		
Stage 1	22.7±0	27.3±0†
Stage 2	2.9±0	3.8±0†
Stage 3	0.6±0	0.8±0†

*p<0.001, †p<0.01, ‡p<0.05
